# Hot Water Extract of *Curcuma longa* L. Improves Serum Inflammatory Markers and General Health in Subjects with Overweight or Prehypertension/Mild Hypertension: A Randomized, Double-Blind, Placebo-Controlled Trial

**DOI:** 10.3390/nu11081822

**Published:** 2019-08-07

**Authors:** Ryusei Uchio, Koutarou Muroyama, Chinatsu Okuda-Hanafusa, Kengo Kawasaki, Yoshihiro Yamamoto, Shinji Murosaki

**Affiliations:** Research & Development Institute, House Wellness Foods Corp., 3-20 Imoji, Itami, Hyogo 664-0011, Japan

**Keywords:** turmeric (*Curcuma longa* L.), bisacurone, turmeronol, chronic inflammation, C-reactive protein, glucose, hemoglobin A1c, high-density cholesterol, short-form 36-item health survey, profile of mood states

## Abstract

To investigate the effect of a hot water extract of *C. longa* L. (WEC) containing anti-inflammatory agents, bisacurone, and turmeronol on chronic inflammation, a randomized double-blind placebo-controlled study was conducted in middle-aged and elderly subjects aged 50–69 years with overweight or prehypertension/mild hypertension. The subjects consumed 900 mg WEC tablets, containing 400 μg bisacurone, 80 μg turmeronol A and 20 μg turmeronol B (WEC group: *n* = 45), or placebo tablets without WEC (placebo group: *n* = 45) daily for 12 weeks. Serum inflammatory and metabolic markers were measured. The subjects also completed the MOS 36-item short-form health survey (SF-36) and the Profile of Mood States scale (POMS). In the WEC group, the serum levels of C-reactive protein, tumor necrosis factor-α, interleukin-6, and soluble vascular cell adhesion molecule-1 decreased significantly. Compared with the placebo group, the WEC group had significantly lower serum levels of glucose, hemoglobin A1c, and triglycerides, as well as higher serum levels of high-density lipoprotein cholesterol. The WEC group also showed significant improvement of SF-36 scores (for general health, vitality, mental health, and mental summary component) and POMS scores for positive mood states (vigor-activity and friendliness). In conclusion, WEC may ameliorate chronic low-grade inflammation, thus contributing to the improvement of associated metabolic disorders and general health.

## 1. Introduction

Acute inflammation is a physiological response to infection or injury that involves vasodilatation and an increase of vascular permeability, recruitment of immune cells to inflamed tissues for targeting pathogenic microorganisms and elimination of dead cells, and initiation of the processes of tissue repair and regeneration [1]. In contrast, chronic inflammation is a persistent inflammatory response mediated by long-lived immune cells that can have various adverse consequences, including tissue damage, inhibition of healing, promotion of fibrosis, and disruption of homeostasis [1]. Recently, low-grade inflammation was recognized as a state in which systemic inflammatory mediators show only slight elevation relative to the levels seen in acute inflammation [2,3]. Although this type of inflammation has not been clearly defined, it is considered to be related to aging [4], obesity [3], hypertension [5], and an unhealthy lifestyle [6] in the absence of obvious infection or tissue injury [2]. Previous reports have indicated that chronic low-grade inflammation is potentially associated with an increased risk of metabolic syndrome [7], atherosclerotic disease [8], Alzheimer’s disease [9], and cancer [10].

Inflammatory mediators, including C-reactive protein (CRP), tumor necrosis factor (TNF)-α, interleukin (IL)-6, and vascular cell adhesion molecule (VCAM)-1, are produced by various cells in response to stimulation by conserved microbial structures, tissue damage signals, and metabolic stress [1,2,3]. These mediators are known to contribute to the development of chronic inflammation and to the occurrence of metabolic abnormalities, including glucose intolerance, insulin resistance, and a pro-atherogenic lipid profile [3,7,11]. Elevation of circulating levels of inflammatory molecules has been reported to possibly be associated with an impaired quality of life (QOL) and with mood disturbance [12,13]. Recently, it was reported that aging is associated with a slight increase in the levels of pro-inflammatory mediators [4]. These inflammatory mediators are known to be involved in the development of various age-related conditions, including low back pain [14], shoulder dysfunction [15], and cognitive impairment [16].

*Curcuma longa*, also known as turmeric, is a member of the Zingiberaceae family, and has traditionally been used as a medicinal herb with various biological activities [17]. Aqueous extracts of turmeric have antioxidant and anti-inflammatory effects [18], promote corneal wound healing [19], show antidepressant activity [20], and also have an anticancer effect [21]. In addition, a hot water extract of *C. longa* (WEC) was reported to prevent various chronic inflammatory diseases in mice, including cotton pellet-induced granuloma [22], carbon tetrachloride-induced hepatitis [23], and non-alcoholic steatohepatitis, by decreasing the expression of genes coding for inflammatory cytokines and cell adhesion molecules [24]. It was also reported that intake of WEC improved the emotional state of healthy subjects [25]. However, the influence of WEC on chronic inflammation in humans is not clearly understood.

To evaluate the effect of WEC on chronic inflammation and associated conditions, we measured the blood levels of inflammatory/metabolic markers and assessed general health using two questionnaires (MOS 36-item short-form health survey (SF-36) and the Profile of Mood States scale (POMS)) in middle-aged and elderly subjects who were considered to be susceptible to chronic inflammation because they were overweight/mildly obese or had prehypertension/mild hypertension.

## 2. Materials and Methods

### 2.1. Study Design

**Figure 1:**
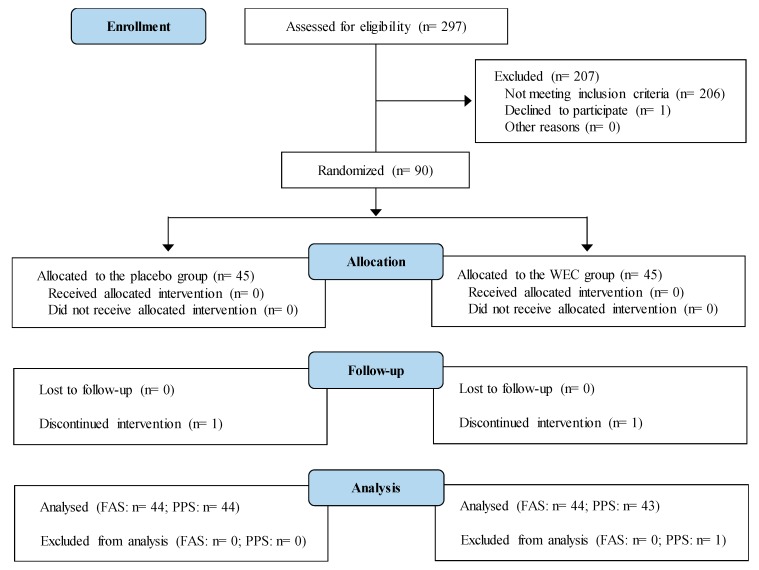
Study flow diagram (CONSORT 2010).

A 12-week, randomized, double-blind, placebo-controlled interventional study was conducted according to the Declaration of Helsinki guidelines. All the procedures involving human participants were approved by the institutional review board of Chiyoda Paramedical Care Clinic (Tokyo, Japan). Prior to enrollment in the study, written informed consent was obtained from all the participants after they read a detailed consent form. This study was performed at the Chiyoda Paramedical Care Clinic from July to December 2017 by a contract research organization (CRO; CPCC Co., Ltd., Tokyo, Japan). The study was registered with the University hospital Medical Information Network (UMIN; Registration number, UMIN000029095). Figure 1 shows a Consolidated Standards of Reporting Trials (CONSORT) 2010 flow diagram of the participants from enrolment to analysis [26]. The completed CONSORT checklist is provided in Table S1.

### 2.2. Enrollment of Participants

Recruitment for the study was conducted from July to September 2017 in Tokyo, Japan. A total of 297 consecutive candidate subjects underwent assessment for eligibility. The inclusion criteria were middle-aged to elderly subjects (aged 50 to 69 years), including men and menopausal women with a body mass index (BMI) between overweight and obesity class 1 (≥23 to <30 kg/m^2^) [27] or blood pressure between prehypertension and grade 1 (mild) hypertension (systolic blood pressure (SBP) ≥120 to <160 mmHg or diastolic blood pressure (DBP) ≥80 to <100 mmHg) [28]. The exclusion criteria were as follows: (1) persons who were positive for hepatitis C virus antibody or hepatitis B surface antigen, (2) persons using medications or health foods that could possibly influence the results of this study, (3) persons with a history of heart disease, liver disease, kidney disease, or gastrointestinal disease, (4) persons with a history of cardiovascular disease, (5) persons with excessive alcohol intake (mean daily consumption of 60 g or more) or smoking (mean daily consumption of two packs), (6) persons with extremely irregular dietary habits, (7) persons with allergies to medications or foods, (8) persons who were participating in another trial, had participated in the past 4 weeks, or planned to participate in another trial during the scheduled study period, (9) persons who had made a blood donation within one month prior to this study, (10) men who had made a blood donation of 400 mL within three months prior to this study, (11) women who had made a blood donation of 400 mL within four months prior to this study, (12) men who had made a blood donation over an amount (1200 mL minus the estimated volume of blood collected during the study) within one year prior to this study, (13) women who had made a blood donation over an amount (800 mL minus the estimated volume of blood collected during the study) within one year prior to this study, (14) persons judged to be unsuitable for the study for other reasons by the investigators.

### 2.3. Study Agent

The composition of the tablets used in this study is described in Table 1. The base tablets were composed of maltose, silicon dioxide, and sucrose esters. Placebo tablets also contained safflower and kaoliang as coloring agents to match the color of the WEC tablets. A hot water extract of *Curcuma longa*, Turmeric Extract (WEC; House Wellness Foods) was added to the WEC tablets. WEC was prepared according to the method described previously [25,29]. In brief, rhizomes of *Curcuma longa* were extracted for 1 h with hot water at 98 °C, after which the supernatant was concentrated, mixed with dextrin, and spray dried to obtain WEC powder.

### 2.4. Intervention

The intervention was conducted from September to December 2017. From among the 297 candidates, the contract research organization selected 90 subjects who satisfied the inclusion/exclusion criteria and had relatively high serum CRP levels. The test tablets and the selected subjects were randomly assigned numbers by the authors and the CRO, respectively, and the assignment list was stored carefully until database locking. Throughout the study, all the subjects and investigators were blinded to the treatment provided. The subjects were randomly allocated to two groups by stratified randomization for sex, age, BMI, blood pressure (SBP and DBP), and CRP. Each subject took three tablets with WEC (WEC group: *n* = 45) or without WEC (placebo group: *n* = 45) once daily for 12 weeks. Subjects visited the study center in weeks 0, 4, 8, and 12 to undergo interviews by an experienced doctor, physiological measurements, hematology and biochemistry tests, urinalysis, and completion of questionnaires. Hematology tests, biochemistry tests, and urinalysis were performed by a contract laboratory company (LSI Medience Co., Ltd., Tokyo, Japan). Evaluation of the questionnaires was conducted by CPCC Co., Ltd. (Tokyo, Japan). The subjects were asked to record the following information in a diary on a daily basis until the end of the study: occurrence of diseases and symptoms; intake of the study tablets, health foods, and medications; and the reasons for taking medications, the dosage, and the duration of use. Subjects were asked to adhere to the following rules until the end of the study: (1) maintaining their usual lifestyle throughout the study period, including diet, exercise, drinking, smoking, and medication; (2) avoiding intake of health foods other than the study tablets; and (3) avoiding excessive drinking, smoking, exercise, fasting, and consumption of an unbalanced diet. Subjects were instructed to fast, except for intake of water, after 9:00 p.m. on the day before the examination.

### 2.5. Measurement of Inflammatory Markers

The serum level of high-sensitive CRP (hsCRP) was measured by an immunonephelometric method with an upper detection limit of 0.500 mg/dL. For samples exceeding this limit, remeasurements were carried out by standard latex agglutination turbidimetry. The serum levels of TNF-α and IL-6 were determined by chemiluminescent enzyme immunoassays, while serum IL-1β and sVCAM-1 levels were measured with enzyme-linked immunoassays.

### 2.6. Short-Form 36 Health Status Questionnaire

The second edition of SF-36 is a widely used self-reported questionnaire comprising 36 items for evaluation of health-related QOL, which has demonstrated good reliability and validity [30,31]. Items are aggregated into the following eight subscales: physical functioning (PF), role physical (RP), bodily pain (BP), general health (GH), vitality (VT), social functioning (SF), role emotional (RE), and mental health (MH). All the items were transformed and summed according to the official manual to give scores from 0 to 100 points for each scale, with higher scores indicating better QOL. The scores for the eight scales were then used to provide scores for two main components, which were the physical component summary (PCS) score and the mental component summary (MCS) score. Three scales (PF, RP, and BP) made a major contribution to the PCS and three other scales (SF, RE, and MH) were the main determinants of the MCS. The remaining two scales (GH and VT) were used for calculating both component summary scores. Then, the sums of the PCS and MCS were each multiplied by 10, and 50 was added to achieve linear transformation to the T-score metric, which has a mean of 50 and a standard deviation of 10 in the general Japanese population.

### 2.7. Profile of Mood States Scale

The second edition of POMS short version is a widely used self-reported questionnaire for rapid assessment of mood states, including transient, fluctuating feelings, and enduring affect states [32,33,34]. POMS contains 35 questions that assess the following seven different moods: anger–hostility (AH), confusion–bewilderment (CB), depression–dejection (DD), fatigue–inertia (FI), tension–anxiety (TA), vigor–activity (VA), and friendliness (F). Subjects were asked to indicate their mood states during the previous one-week period on a five-point scale ranging from “not-at-all” to “extremely”. Total mood disturbance (TMD) scores were calculated for each subscale by the following formula: TMD = AH + CB + DD + FI + TA − VA. High scores for VA and F indicate a positive mood state, while high scores for AH, CB, DD, FI, TA, and TMD indicate a negative mood state.

### 2.8. Sample Size

To calculate the minimum number of subjects required for adequate statistical power, we used the G Power 3.1.9 program (University of Dusseldorf, Dusseldorf, Germany) [35]. In a previous clinical study, a green tea extract with anti-inflammatory activity reduced the serum CRP level by about 30% from baseline [36]. A sample size of 45 subjects per group was estimated to be sufficient for the present study based on the following assumptions: 30% reduction of serum CRP by WEC, Cohen’s d value = 0.60, statistical power = 80%, and type I error = 5% (two-tailed).

### 2.9. Statistical Analysis

Statistical analysis was performed using the intention-to-treat (ITT) population, which was defined as all randomized subjects and the full analysis set (FAS) for assessment of safety, while the per-protocol set (PPT) was used for evaluation of efficacy. In the efficacy assessment of CRP, we excluded the data of subjects who had serum CRP > 0.3 mg/dL, which is generally accepted as indicating acute inflammation in Japan [37], and who had symptoms associated with acute inflammation as judged by physicians (common cold symptoms—cough, nasal discharge, pharyngeal itching/pain, headache, chills, fatigue, arthralgia, and/or fever—myalgia, or sprains). All statistical analyses were performed with the IBM SPSS statistical software package (version 25) for Windows (IBM Corp., New York, NY, USA). The results are presented as the mean ± standard deviation (SD). Comparison of adverse events between the placebo group and the WEC group was done with Fisher’s exact test. Baseline characteristics were compared between the two groups by the two-tailed unpaired Student’s *t*-test when variance was homogeneous or the Aspin-Welch *t*-test when variance was heterogeneous, except for the results of the urinalysis, which were analyzed by the two-tailed Mann–Whitney *U*-test. Changes from baseline were analyzed by repeated measure two-way ANOVA (two groups × three time points) with the SPSS general linear model for determining the main effects of group and time, and their interaction, followed by comparison between the placebo and WEC groups at each time point using simple main effect tests [38,39,40]. A probability (*p*) value < 0.05 was considered to indicate statistical significance.

## 3. Results

### 3.1. Subjects

The disposition of the subjects is shown in Figure 1. A total of 90 out of 297 candidates were randomly allocated to the placebo group or the WEC group (45 per group). Two participants dropped out before completing the study, including one with cerebral infarction in the placebo group and one with ovarian cystectomy in the WEC group. The other 88 subjects completed the study. However, one subject in the WEC group had a test agent intake rate of 90% and did not comply with the protocol, and was therefore excluded from efficacy analysis. Baseline characteristics showed no significant differences between the WEC group and the placebo groups (Table 2 and Table 3). In addition, the average test agent intake rate was not significantly different between the WEC group (99.3 (SD 1.8)%) and the placebo group (99.3 (SD 1.2)%).

### 3.2. Effect of WEC on Serum Inflammatory Markers

The change of CRP from baseline tended to be lower in the WEC group than in the placebo group throughout the study period (*p* = 0.077 by repeated measures ANOVA (r-ANOVA)), and it was significantly lower in weeks 8 and 12 in the WEC group compared with the placebo group (Table 4). The changes of TNF-α and IL-6 from baseline showed no significant differences between the two groups throughout the study period (r-ANOVA), but they were significantly lower in weeks 4, 8, and 12 in the WEC group compared with the placebo group (Table 4). The decrease of sVCAM-1 from baseline was significantly larger in weeks 4 and 12 in the WEC group compared with the placebo group (Table 4). Changes of IL-1β from baseline showed no significant differences between the two groups (Table 4).

### 3.3. Effect of WEC on Serum Metabolic Markers

The changes in glucose levels from baseline tended to be lower in the WEC group than in the placebo group throughout the study period (*p* = 0.070, r-ANOVA), and were significantly lower in weeks 4, 8, and 12 in the WEC group compared with the placebo group (Table 5). The change of HbA1c from baseline in week 12 was significantly lower in the WEC group compared with the placebo group (Table 5). In addition, the change of TG from baseline in week 8 was significantly lower in the WEC group than in the placebo group (Table 5). The changes of HDL-cholesterol from baseline tended to be higher in the WEC group than in the placebo group throughout the study period (*p* = 0.099, r-ANOVA), and it was significantly higher in week 8 in the WEC group compared with the placebo group (Table 5). WEC had no effects on the levels of total-cholesterol and LDL-cholesterol (Table 5).

### 3.4. Effect of WEC on SF-36 Scores

Table 6 displays the SF-36 scores. The changes of GH scores from baseline were significantly higher in the WEC group than in the placebo group over the study period (r-ANOVA), and they were significantly higher in weeks 8 and 12 in the WEC group compared with the placebo group. The changes of the VT and MH scores from baseline in week 12 were significantly higher in the WEC group than the placebo group. On the other hand, the changes of the SF and RE scores from baseline in week 8 were significantly lower in the WEC group compared with the placebo group. The change of the PCS score from baseline in week 8 was significantly lower in the WEC group than the placebo group. In contrast, changes of the MCS score from baseline tended to be higher in the WEC group than in the placebo group (*p* = 0.079, r-ANOVA) throughout the study period, and they were significantly higher in weeks 8 and 12 in the WEC group compared with the placebo group.

### 3.5. Effect of WEC on POMS Scores

The change of the VA score from baseline in week 12 was significantly higher in the WEC group than in the placebo group (Table 7). The change of the *F* score from baseline in week 12 was also significantly higher in the WEC group, compared with the placebo group (Table 7).

### 3.6. Safety

Assessment of side effects and adverse effects was conducted in the ITT population (placebo group: *n* = 45; WEC group: *n* = 45). None of the participants reported any side effects. A total of 31 adverse events were recorded throughout the study, with no significant difference in the number of adverse events between the placebo group (15 events) and the WEC group (16 events). The following adverse events occurred in the placebo group: four cases of common cold symptoms, one case of low back pain, one case of colonic polypectomy, one case of cerebral infarction (loss of fine movement of the right index finger and thumb), one case each of increased γ-GTP and uric acid levels, two cases of increased TG and CRP levels, three cases of increased CPK, and two cases of positive urinary protein. The following adverse events occurred in the WEC group: five cases of common cold symptoms, one case of an ankle sprain, one case of bronchitis-like symptoms, one case of mouth ulcers, one case of ovarian cystectomy, three cases of increased CRP, one case each of increased white blood cell count, TG, CPK, and uric acid levels, and two cases of positive urinary protein. These adverse events were mild and were judged to be unrelated to the dietary intervention by an experienced physician. Assessment of safety parameters was performed in the FAS population (placebo group: *n* = 44, WEC group: *n* = 44). Among male subjects, the level of aspartate aminotransferase in week 8 and creatinine in week 4 was significantly higher in the WEC group than in the placebo group (*p* < 0.05), but these changes were within the corresponding reference ranges. The results of other hematology and biochemistry tests, urinalysis, and physiological tests did not differ significantly between the two groups.

## 4. Discussion

The present 12-week, randomized, double-blind, placebo-controlled study was performed to investigate the effect of WEC on chronic inflammation, metabolic parameters, and general health in middle-aged to elderly subjects with overweight or prehypertension/mild hypertension. We found that intake of WEC not only improved serum inflammatory markers (CRP, TNF-α, IL-6, and sVCAM-1), but also reduced the serum levels of glucose, HbA1c, and triglycerides, while increasing HDL-cholesterol levels. In addition, SF-36 scores (GH, VT, MH, and MCS) and POMS scores (VA and F) were significantly higher in the WEC group than in the placebo group. These results suggest that daily intake of WEC could potentially suppress chronic inflammation and improve carbohydrate/lipid metabolism, as well as improving health-related QOL and positive mood states.

CRP is a marker of systemic inflammation that has been reported to show seasonal variation, being higher during winter than during summer [41,42]. In the present study, we observed that the serum CRP level increased from baseline (summer) to week 12 (winter) (Table 4). In recent years, slight elevation of CRP, which cannot be detected by the standard CRP assay, has been accurately quantitated by the hsCRP assay as a marker of low-grade inflammation [2,3]. A slight elevation of hsCRP (about 0.12 mg/dL) was reported to be associated with an increased risk of cardiovascular disease [8], coronary heart disease [43], myocardial infarction [44], diabetes [45], and colon cancer [10]. These diseases are also associated with several inflammatory mediators, such as TNF-α, IL-6, and VCAM-1 [1,46], and their risk may be reduced by anti-inflammatory agents [47,48,49]. In the present study, WEC significantly reduced the serum levels of CRP, TNF-α, IL-6, and sVCAM-1 in middle-aged and elderly subjects with overweight or prehypertension/mild hypertension (Table 4). These results suggest that WEC may ameliorate low-grade inflammation associated with aging, obesity, or hypertension, and thus, could possibly decrease the risk of chronic inflammatory diseases.

Low-grade inflammation is triggered by several factors, including an excessive intake of nutrients (free fatty acids and glucose) [6], endoplasmic reticulum stress [2], and damage-associated molecular patterns [3]. These triggers have been reported to activate the nuclear transcription factor kB (NF-kB) signaling pathway, thus promoting the expression of pro-inflammatory mediators [2,3,6]. TNF-α and IL-6 are both targets of NF-kB and are mainly produced by monocytes/macrophages, and are known to increase hepatic production of CRP and expression of leucocyte/endothelial cell adhesion proteins such as VCAM-1 by endothelial cells [1,29]. CRP promotes differentiation of human monocytes to a pro-inflammatory M1 phenotype [50] and upregulates VCAM-1 expression by endothelial cells [51]. Previous studies have shown that WEC suppresses TNF-α-induced phosphorylation of I kappa B-alpha (IkBα), which can lead to activation of NF-kB, and also inhibits hepatic expression of TNF-α, IL-6, and VCAM-1 mRNA and recruitment of monocytes/macrophages in animal models of nonalcoholic steatohepatitis [24,29]. In addition, WEC was reported to inhibit the elevation of circulating TNF-α and IL-6 levels and ameliorate various chronic inflammatory diseases in animal models, including cotton pellet-induced granuloma [22] and carbon tetrachloride- and γ-irradiation-induced hepatitis [23,52]. Some components of WEC have been reported to show anti-inflammatory activity. For example, bisacurone suppresses VCAM-1-dependent adhesion of monocytes to TNF-α-stimulated human endothelial cells [53]. Recently, turmeronol A and B isolated from WEC were found to inhibit production of inflammatory mediators, including prostaglandin E_2_ and nitric oxide, by activated macrophages [54]. Therefore, it is possible that WEC reduces the production of inflammatory mediators, hepatic infiltration of monocytes/macrophages, and production of CRP, resulting in the suppression of low-grade inflammation.

Metabolic syndrome is defined as the presence of three or more of the following risk factors: visceral obesity, high blood pressure, high fasting glucose, low HDL-cholesterol, and high triglycerides. This metabolic disorder is associated with an increased risk of type 2 diabetes and cardiovascular disease [55]. Tamakoshi et al. reported that slight elevation of CRP, indicating low-grade systemic inflammation, was associated with an increase in the number of risk factors for metabolic syndrome [7]. Dysregulation of carbohydrate/lipid metabolism can be induced by pro-inflammatory cytokines, such as TNF-α and IL-6, both of which inhibit insulin-induced glucose uptake by suppressing the insulin receptor substrate 1/phosphatidylinositol 3 kinase signaling pathway [3]. In addition, pro-inflammatory cytokines promote hepatic lipid accumulation by increasing fatty acid uptake, reducing fatty acid oxidation, and enhancing triglyceride synthesis in the liver [56]. Inflammation also leads to an increase in the production of HDL cholesterol-degrading enzymes, including secretory phospholipase A2 and matrix metalloproteinases [11]. A clinical study showed that anti-inflammatory therapy improved glucose metabolism and markers of lipid metabolism, such as triglycerides and HDL-cholesterol [11,48]. In an obese mouse model, supplementation with herb mixtures containing WEC decreased serum inflammatory cytokine levels and improved hyperglycemia and atherogenic lipid profile such as high triglycerides and low HDL-cholesterol via the inhibition of mRNA expression of hepatic fatty acid synthase and sterol regulatory element binding proteinsi-1c [57,58]. In the present study, intake of WEC significantly reduced serum glucose and HbA1c levels, as well as improving triglyceride and HDL-cholesterol levels (Table 5). These results suggest that WEC could potentially be used to treat the metabolic disorders caused by low-grade chronic inflammation.

According to the World Health Organization, QOL is defined as individual’s perception of their position in life embedded in a cultural, social, and environmental context [59]. Health-related QOL (HR-QOL) can be evaluated by the self-reported SF-36 questionnaire, which covers several domains including physical and psychological health. In the present study, WEC significantly improved the SF-36 subscale scores for general health (GH), vitality (VT), and mental health (MH), and also increased the mental component summary (MCS) score (Table 6). Chronic low-grade inflammation may be related to impairment of HR-QOL [12]. Inflammation is known to reduce cellular energy levels and to induce central nervous system inflammation and circadian dysfunction, leading to the onset of symptoms such as fatigue, depression, sleep disturbance, cognitive impairment, and pain [60,61,62]. Middle-aged persons with fatigue have reportedly shown low scores for GH and VT (commonly used to evaluate fatigue), along with elevations of plasma CRP and IL-6 levels [63]. In addition, high baseline levels of these inflammatory markers were shown to predict fatigue (low VT scores) in a follow-up study [64]. Furthermore, elevation of TNF-α and IL-6 levels was associated with a lower VT score, as well as a lower score for MH (which is used for detection of depression) in subjects with pulmonary hypertension [65,66]. In previous clinical studies, supplementation with anti-inflammatory medicinal herbs improved the scores for GH, VT, MH, and MCS [67,68]. WEC have been found to prevent not only the production of TNF-α and IL-6 in activated brain microglial cells/macrophages, but also neuroinflammation associated with fatigue, depression, and memory impairment in animal studies [20,69,70]. Therefore, it is possible that WEC improved HR-QOL by alleviating inflammation-related symptoms.

POMS is a widely used self-reported questionnaire that measures seven mood states (including negative and positive moods), as well as mood disturbance or emotional distress. In the present study, intake of WEC significantly increased the positive POMS mood scores, which are vigor-activity (VA) and friendliness (F) (Table 7). These positive mood states are known to be influenced by inflammation. For example, systemic low-grade inflammation induced by low-dose infusion of endotoxin was shown to increase blood levels of CRP, TNF-α, and IL-6 in healthy subjects, and also induce sleep disturbance, as well as decreasing the VA POMS score [13,71,72]. Chronic sleep disturbance is known to reduce the cortical oxygenation response and promote mood disturbance, as detected by a low VA score [73]. Using the Trier Social Stress Test, tasks that induce psychological stress were associated with an elevated systemic IL-6 level and a decrease of the POMS F score [74,75]. Moreover, a clinical study has shown that anti-inflammatory foods improve the scores for positive mood states (VA and F) [76,77]. *C. longa* was reported to have the potential to reduce the production of inflammatory mediators such as nitric oxide and ameliorate anxiety- and sleep deprivation-induced behavior abnormalities in a stressed animal [78,79]. Therefore, WEC may increase the POMS scores for positive mood states by reducing the levels of inflammatory mediators.

## 5. Conclusions

In conclusion, we conducted a 12-week, randomized, double-blind, placebo-controlled study in middle-aged and elderly subjects with overweight or prehypertension/mild hypertension. Compared with the placebo group, the serum levels of inflammatory mediators, glucose, HbA1c, and triglycerides were significantly lower, whereas HDL-cholesterol was significantly higher in the WEC group. In addition, the WEC group showed significant improvement of HR-QOL (SF-36) and positive mood states assessed by POMS. These results suggest that intake of a hot water extract of *Curcuma longa* may have the potential to improve general health by reducing chronic low-grade inflammation.

## Figures and Tables

**Table 1:** Composition of the study tablets (per three tablets).

	Placebo (0.9 g/3 Tablets)	WEC (0.9 g/3 Tablets)
Energy (kcal)	3.4	3.4
Carbohydrate (g)	0.820	0.760
Protein (g)	0	0.019
Lipid (g)	0.013	0.031
Sodium chloride (mg)	0.74	0.45
Bisacurone (μg)	0	400
Turmeronol A (μg)	0	80
Turmeronol B (μg)	0	20

**Table 2:** Baseline characteristics of the subjects with potential inflammation ^1^.

	Placebo Group (*n* = 44)		WEC Group (*n* = 43)		*p* Value
	Mean	SD	Mean	SD	
Sex (*n*, Male/Female)	22/22		23/20		0.746
Age (years)	58.5	5.5	58.8	5.3	0.803
Physical measurements and tests					
BMI (kg/m^2^)	25.1	2.5	25.0	1.8	0.561
SBP (mmHg)	131.0	16.8	129.2	15.3	0.626
DBP (mmHg)	82.4	11.6	81.6	10.5	0.642
Serum inflammatory markers					
CRP (mg/dL)	0.105	0.081	0.090	0.064	0.354
TNF-α (pg/mL)	1.557	0.358	1.936	1.945	0.215
IL-1β (pg/mL)	0.057	0.091	0.051	0.102	0.771
IL-6 (pg/mL)	1.125	0.412	4.381	11.566	0.072
sVCAM-1 (ng/mL)	571.0	121.7	599.3	129.7	0.297
Metabolic markers					
Glucose (mg/dL)	84.2	6.0	86.0	6.9	0.178
HbA1c (%)	5.48	0.24	5.51	0.28	0.535
Triglyceride (mg/dL)	112.5	56.2	127.5	74.1	0.272
Total cholesterol (mg/dL)	220.1	38.6	218.3	33.5	0.778
LDL-cholesterol (mg/dL)	138.1	34.2	132.8	29.9	0.384
HDL-cholesterol (mg/dL)	54.3	14.9	56.0	12.3	0.579

BMI: body mass index; DBP: diastolic blood pressure; HbA1c: hemoglobin A1c; HDL: high-density lipoprotein; CRP: C-reactive protein; IL-1β: interleukin-1β; IL-6: interleukin-6; LDL: low-density lipoprotein; SBP: systolic blood pressure; sVCAM-1: soluble vascular cell adhesion molecule-1; TNF-α: tumor necrosis factor-α. ^1^ Values are the mean and standard deviation for *n* = 44 (placebo group) or *n* = 43 (WEC group). Comparison of sex was performed with the two-tailed Mann–Whitney *U* test. Comparisons of physical measurements, serum inflammatory markers, and biochemical data were performed with the unpaired Student’s *t*-test when variance was homogeneous or with the unpaired Aspin-Welch *t*-test when variance was heterogeneous.

**Table 3:** Baseline scores for the MOS 36-item short-form health survey (SF-36) and Profile of Mood States (POMS) questionnaire ^1^.

	Placebo Group (*n* = 44)		WEC Group (*n* = 43)		*p* Value
	Mean	SD	Mean	SD	
SF-36 scores (points)					
Physical functioning (PF)	91.7	8.8	94.3	6.5	0.121
Role physical (RP)	91.3	14.3	95.6	11.0	0.120
Bodily pain (BP)	78.7	16.3	78.5	17.6	0.958
General health (GH)	74.5	15.9	74.8	13.6	0.910
Vitality (VT)	66.3	20.2	67.2	14.2	0.828
Social functioning (SF)	90.3	18.7	94.8	11.6	0.189
Role emotional (RE)	90.2	15.5	93.2	11.4	0.295
Mental health (MH)	77.2	13.4	79.0	13.5	0.536
Physical component summary (PCS)	51.0	7.9	53.3	5.2	0.117
Mental component summary (MCS)	53.5	8.3	53.6	6.9	0.951
POMS scores (points)					
Anger–hostility (AH)	46.9	7.1	47.1	5.9	0.896
Confusion–bewilderment (CB)	47.9	8.2	47.2	9.1	0.716
Depression–dejection (DD)	47.4	5.5	46.7	6.7	0.602
Fatigue–inertia (FI)	47.6	8.3	44.8	6.1	0.077
Tension–anxiety (TA)	48.0	9.5	46.8	9.2	0.563
Vigor–activity (VA)	56.3	10.3	56.1	9.3	0.932
Friendliness (F)	56.8	9.3	56.1	10.1	0.770
Total mood disturbance (TMD)	45.8	8.2	44.6	7.8	0.496

^1^ Values are the mean with standard deviations for *n* = 44 (placebo group) or *n* = 43 (WEC group). Comparisons of SF-36 and POMS data were performed with the unpaired Student’s *t*-test when variance was homogeneous or the unpaired Aspin-Welch *t*-test when variance was heterogeneous.

**Table 4:** Effect of a hot water extract of *Curcuma longa* (WEC) on serum inflammatory markers ^1^.

	Change from Baseline						Repeated Measures Two-Way ANOVA		
	Week 4		Week 8		Week 12		Group	Time	Interaction
	Mean	SD	Mean	SD	Mean	SD			
CRP (mg/dL)									
Placebo	0.016	0.072	0.036	0.092	0.057	0.138	0.077	0.098	0.243
WEC	0.006	0.086	−0.001*	0.071	0.015 **	0.068			
TNF-α (pg/mL)									
Placebo	0.718	0.335	0.824	0.448	0.215	0.342	0.165	< 0.001	0.696
WEC	0.381 **	1.806	0.410 **	1.754	−0.153 **	1.718			
IL-1β (pg/mL)									
Placebo	0.013	0.105	0.029	0.110	0.019	0.120	0.474	0.270	0.020
WEC	0.067	0.144	0.010	0.120	0.033	0.159			
IL-6 (pg/mL)									
Placebo	0.21	1.41	0.49	1.12	0.33	0.70	0.150	0.042	0.021
WEC	−2.26 **	10.32	−2.41 **	10.91	−1.33 **	11.09			
sVCAM-1 (mg/dL)									
Placebo	12.7	85.9	6.1	59.6	33.5	59.9	0.112	0.024	0.780
WEC	−14.1 *	92.7	−10.3	98.9	5.9 *	95.7			

CRP: C-reactive protein; IL: interleukin; sVCAM-1: soluble vascular cell adhesion molecule-1; TNF-α: tumor necrosis factor-α. ^1^ Values are the mean with standard deviation for *n* = 44 (placebo group) or *n* = 43 (WEC group). * *p* < 0.05, ** *p* < 0.01: Significant difference from the placebo group by repeated measures two-way ANOVA, followed by a simple main effect test.

**Table 5:** Effect of a hot water extract of *Curcuma longa* (WEC) on serum metabolic markers ^1^.

	Change from Baseline						Repeated Measures Two-Way ANOVA		
	Week 4		Week 8		Week 12		Group	Time	Interaction
	Mean	SD	Mean	SD	Mean	SD			
Glucose (mg/dL)									
Placebo	2.2	4.6	2.7	4.7	2.0	5.0	0.070	0.153	0.900
WEC	0.1 **	6.5	1.0 *	5.5	−0.1 **	6.6			
HbA1c (%)									
Placebo	0.132	0.096	0.018	0.108	0.016	0.103	0.174	< 0.001	0.174
WEC	0.128	0.093	−0.005	0.097	−0.033 **	0.125			
Triglycerides (mg/dL)									
Placebo	15.8	72.4	14.0	55.5	−4.0	47	0.135	0.005	0.784
WEC	3.1	36.8	2.0 *	50.1	−12.6	54.8			
Total cholesterol (mg/dL)									
Placebo	5.5	15.1	2.3	18.2	3.5	15.1	0.751	0.506	0.635
WEC	4.0	16.6	3.8	15.8	2.0	17.8			
LDL−cholesterol (mg/dL)									
Placebo	1.5	18.2	0.6	18.0	2.5	13.3	0.911	1.000	0.619
WEC	2.1	14.6	2.0	16.3	1.4	14.3			
HDL−cholesterol (mg/dL)									
Placebo	1.4	5.1	1.3	5.8	2.8	4.5	0.099	0.022	0.155
WEC	2.1	5.0	4.3 **	5.5	4.2	8.6			

HbA1c: hemoglobin A1c; LDL: low-density lipoprotein; HDL: high-density lipoprotein. ^1^ Values are the mean with standard deviation for *n* = 44 (placebo group) or *n* = 43 (WEC group). * *p* < 0.05, ** *p* < 0.01: Significant difference from the placebo group by repeated measures two-way ANOVA, followed by a simple main effect test.

**Table 6:** Effect of a hot water extract of *Curcuma longa* (WEC) on the MOS 36-item short-form health survey (SF-36) scores ^1^.

	Change from Baseline						Repeated Measures Two-Way ANOVA		
	Week 4		Week 8		Week 12		Group	Time	Interaction
	Mean	SD	Mean	SD	Mean	SD			
Physical functioning (PF)									
Placebo	0.11	5.23	−0.80	5.49	−0.80	7.85	0.674	0.182	0.571
WEC	−0.23	5.87	−2.21	10.02	−0.58	6.83			
Role physical (RP)									
Placebo	−0.43	10.56	1.56	9.82	−0.71	16.68	0.696	0.345	0.267
WEC	−2.33	11.41	−0.58	9.53	1.16	8.10			
Bodily pain (BP)									
Placebo	1.43	17.91	−2.09	17.61	−0.95	19.36	0.475	0.177	0.829
WEC	3.00	17.28	−0.14	19.96	2.67	17.13			
General health (GH)									
Placebo	−2.5	8.55	−4.05	8.83	−3.61	9.90	0.047	0.858	0.503
WEC	−0.05	11.60	0.65 **	10.45	0.09 **	9.36			
Vitality (VT)									
Placebo	−0.14	14.36	−1.28	11.95	−2.27	15.61	0.153	0.820	0.176
WEC	1.16	12.29	1.16	9.38	3.78 **	11.74			
Social functioning (SF)									
Placebo	2.84	15.69	2.84	14.23	−1.42	25.46	0.511	0.950	0.008
WEC	−1.74	16.27	−2.62 *	13.79	2.62	11.43			
Role emotional (RE)									
Placebo	1.89	13.31	2.65	11.33	−0.19	12.90	0.415	0.939	0.127
WEC	−0.19	11.57	−1.55*	11.39	0.97	11.67			
Mental health (MH)									
Placebo	2.16	8.79	−1.02	9.74	−0.91	13.17	0.300	0.085	0.062
WEC	1.98	9.83	0.81	10.06	3.60 **	11.56			
Physical component summary (PCS)									
Placebo	0.17	4.64	0.86	4.10	−0.21	8.69	0.234	0.963	0.247
WEC	−0.97	4.65	−1.42*	6.42	−0.31	4.59			
Mental component summary (MCS)									
Placebo	0.47	5.45	−1.06	4.70	−1.16	7.32	0.079	0.228	0.064
WEC	1.03	5.43	0.71*	5.27	1.99 **	5.08			

^1^ Values are the mean with standard deviations for *n* = 44 (placebo group) or *n* = 43 (WEC group). * *p* < 0.05, ** *p* < 0.01: Significant difference from the placebo group by repeated measures two-way ANOVA, followed by a simple main effect test.

**Table 7:** Effect of a hot water extract of *Curcuma longa* (WEC) on the Profile of Mood States (POMS) scores ^1^.

	Change from Baseline						Repeated Measures Two-Way ANOVA		
	Week 4		Week 8		Week 12		Group	Time	Interaction
	Mean	SD	Mean	SD	Mean	SD			
Anger−hostility (AH)									
Placebo	−1.86	4.64	−1.91	6.45	−2.05	9.20	0.412	0.378	0.482
WEC	−1.09	5.27	−0.16	4.63	−2.07	5.32			
Confusion−bewilderment (CB)									
Placebo	0.18	5.62	−0.16	5.85	0.16	8.95	0.667	0.928	0.751
WEC	−0.35	5.40	−0.05	5.98	−0.84	6.18			
Depression−dejection (DD)									
Placebo	−1.32	3.84	−0.77	4.98	−0.34	7.76	0.616	0.602	0.591
WEC	−0.58	4.72	0.09	5.10	−0.65	3.96			
Fatigue−inertia (FI)									
Placebo	−1.25	5.36	−2.48	6.10	−2.61	7.86	0.486	0.247	0.548
WEC	−1.19	4.61	−0.91	5.84	−2.23	5.23			
Tension−anxiety (TA)									
Placebo	−0.80	5.97	−0.61	6.91	−0.91	7.73	0.976	0.641	0.874
WEC	−0.6	5.90	−0.35	5.96	−1.26	6.88			
Vigor−activity (VA)									
Placebo	−1.64	6.85	−1.52	7.05	−2.14	7.88	0.225	0.044	0.007
WEC	−2.21	7.25	−0.07	7.89	1.91*	7.28			
Friendliness (F)									
Placebo	−0.86	5.99	−1.36	6.92	−1.23	7.74	0.284	0.238	0.089
WEC	−1.21	6.92	0.14	6.82	1.51*	6.06			
Total mood disturbance (TMD)									
Placebo	−0.75	4.02	−1.02	4.89	−0.77	8.85	0.990	0.289	0.206
WEC	−0.26	4.13	−0.30	4.53	−2.02	4.76			

^1^ Values are the mean with standard deviations for *n* = 44 (placebo group) or *n* = 43 (WEC group). * *p* < 0.05: Significant difference from the placebo group by repeated measures two-way ANOVA, followed by a simple main effect test.

## Supplementary Materials

The following are available online at https://www.mdpi.com/2072-6643/11/8/1822/s1, Table S1: CONSORT 2010 checklist of information to include when reporting a randomized trial.
